# Artificial neural networks for the prediction of peptide drift time in ion mobility mass spectrometry

**DOI:** 10.1186/1471-2105-11-182

**Published:** 2010-04-11

**Authors:** Bing Wang, Steve Valentine, Manolo Plasencia, Sriram Raghuraman, Xiang Zhang

**Affiliations:** 1 Department of Electronics and Information Engineering, Anhui University of Technology, Ma'anshan, 243002, China; 2 Department of Chemistry, Center for Regulatory and Environmental Analytical Metabolomics, University of Louisville, Louisville, KY 40292, USA; 3 Department of Chemistry, Indiana University, Bloomington, IN 47405, USA; 4 Predictive Physiology and Medicine Inc. Bloomington, IN 47403, USA

## Abstract

**Background:**

There is an increasing usage of ion mobility-mass spectrometry (IMMS) in proteomics. IMMS combines the features of ion mobility spectrometry (IMS) and mass spectrometry (MS). It separates and detects peptide ions on a millisecond time-scale. IMS separates peptide ions based on drift time that is determined by the collision cross-section of each peptide ion in a given experiment condition. A peptide ion's collision cross-section is related to the ion size and shape resulted from the peptide amino acid sequence and their modifications. This inherent relation between the drift time of peptide ion and peptide sequence indicates that the drift time of peptide ions can be used to infer peptide sequence and therefore, for peptide identification.

**Results:**

This paper describes an artificial neural networks (ANNs) regression model for the prediction of peptide ion drift time in IMMS. Each peptide in this work was represented using three descriptors (i.e., molecular weight, sequence length and a two-dimensional sequence index). An ANN predictor consisting of four input nodes, three hidden nodes and one output node was constructed for peptide ion drift time prediction. For the model training and testing, a 10-fold cross-validation strategy was employed for three datasets each containing different charge states. Dataset one contains 212 singly-charged peptide ions, dataset two has 306 doubly-charged peptide ions, and dataset three has 77 triply-charged peptide ions. Our proposed method achieved 94.4%, 93.6% and 74.2% prediction accuracy for singly-, doubly- and triply-charged peptide ions, respectively.

**Conclusions:**

An ANN-based method has been developed for predicting the drift time of peptide ions in IMMS. The results achieved here demonstrate the effectiveness and efficiency of the prediction model. This work can enhance the confidence of protein identification by combining with current database search approaches for protein identification.

## Background

Proteomics is the large-scale identifying and quantifying all expressed proteins in biological samples. Understanding protein expression, the structure and function of each protein and the interactions among them will facilitate the search of useful targets and biomarkers for pharmaceutical design. Currently, mass spectrometry (MS) is an indispensable tool for protein identification and quantification [1-6]. The typical procedures in proteomics include digestion of the protein mixture into peptides, peptide separation using multidimensional liquid chromatography (MDLC), and finally MS for quantification and tandem mass spectrometry (MS/MS) for identification of proteins from which the peptides were derived [7-10].

Ion mobility-mass spectrometry (IMMS), which combines the features of ion mobility spectrometry (IMS) and MS, separates and detects peptide ions on a millisecond time-scale [11-13]. A typical proteomics experimental setup using IMMS consists of five components: sample introduction, compound ionization, ion mobility separation, mass separation as well as peptide and protein ion detection [14]. Firstly, peptides mixture is introduced into the IMMS system. All peptides are ionized by electrospray ionization (EI). The ionized peptide ions are then subjected to a drift tube for separation based on the mobility of peptide ions. The separated peptide ions are further submitted to an MS, where peptide ions are separated by mass-to-charge ratio (m/z) and finally detected by a mass detector. Although these five components all play essential roles in the process, ion mobility separation is crucial for its impact on the consequent mass analysis and peptide ion detection. Ion mobility allows for the separation of peptide ions based on differing cross-sections and molecular charge. This advantage makes it possible for peptides with the same mass-to-charge (m/z) ratio to be discriminated by the difference of their cross-section-to-charge ratio. To achieve high confidence peptide identification, many researchers have enhanced peptide ion separation based on changing the ion mobility conditions such as employing different gases, altering electric field strengths, and adopting non-linear electric field gradients [15-21]. Even though these efforts, attempting to change the experimental environment, can impact the observed results and improve the ability of IMMS instruments to separate peptides, they are time-consuming and may be difficult to reproduce with different instrumentation configurations.

IMS separates peptide ions based on drift time that is determined by the collision cross-section of each peptide ion in a given experiment condition. A peptide ion's collision cross-section is related to the ion size and shape resulted from the peptide amino acid sequence and its chemical modifications. Therefore, peptide ion drift time in IMS is actually correlated to the peptide amino acid sequence. Deciphering such inherent relation between the drift time of peptide ion and peptide sequence will significantly benefit not only the understanding of gas phase peptide chemistry, but also the identification of peptide and proteins in proteomics.

Peptide selection, fractionation and separation on chromatographic columns may be modelled with various methods. We have developed an algorithm to predict the fractionation of peptides in strong anion exchange (SAX) chromatography using a pattern classification technique based on artificial neural network (ANN) method [22,23]. An ANN has also been used to predict peptide separation in reversed phase (RP) chromatography [24,25]. Predicted peptide retention times have been applied to assist peptide identification [26]. Like other chromatographic methods, IMMS separates peptides based on their chemical and physical properties that reflect the peptide amino acid sequence and associated chemical modifications. It has been reported that the measurement of peptide ion drift time using IMMS is very reproducible [27]. Any two measurements of mobilities (or cross-sections) recorded on the same instrument usually agree to within 1% relative uncertainty. Measurements performed by different groups usually agree to within 2% [28]. The high reproducibility of IMS measurements encouraged us to explore the possibility of predicting peptide ion drift time using commercial IMMS instrumentation. The predicted peptide ion drift time can be used to simulate peptide separation in IMMS and also can be used to enhance confidence in protein identifications.

In this paper, a computational method was proposed to predict peptide ion drift time in IMMS using an artificial neural networks (ANNs) regression model. In seeking a general property to estimate drift time of peptide ions in IMMS, sequence-based information was first extracted from each peptide, including peptide molecular weight, sequence length and a two-dimensional sequence index. The two-dimensional sequence index has two parameters designed to reflect the peptide amino acid sequence information based on the ionization constant (pKa) values of 20 amino acids. Thereafter, a 10-fold cross-validation strategy was employed for ANN model training and testing using three datasets with different charge state assignments. The developed ANN model was tested on a five-protein digest sample. The high prediction accuracy achieved in this work demonstrated the effectiveness and efficiency of the prediction model.

## Results and discussion

In this study we used the peptides generated from tryptic digestion of 20 pure proteins for our model development and testing. The raw data obtained from the mass spectrometer were first processed using instrument control software (MassLynx V4.1) to determine the drift time of each peptide ion. Peptide charge status was manually assigned based on the m/z spacing between isotopic peaks. Peptide ion assignment was achieved using a peptide mass fingerprint approach in which peptide ion assignment thresholds of ±0.02 Da were used. We assigned 595 peptide ions to the 20 proteins. Of these assigned peptide ions, 212 were singly charged, 306 were doubly charged and 77 were triply charged.

To evaluate our proposed method objectively, we randomly grouped the 20 pure proteins into two subsets, {P1} and {P2}, where {P1} contains 15 proteins while {P2} contains 5 proteins. All peptides digested from the protein subset {P1} were used as the training dataset for the ANN model construction, training and cross-validation (CV). The peptides digested from the protein subset {P2} were used for model generalization testing. The motivation of generalization testing is to verify the generalization capability of the trained ANN model using a new dataset which was not used for the creation and training of the network. Table 1 summarizes the numbers of (and types of) all peptide ions identified from the 20 proteins. We then grouped these peptides and corresponding drift times into three datasets, C1, C2 and C3, based on peptide charge states. We have identified 513 peptide ions from the digests of protein subset {P1} and 82 peptide ions from protein subset {P2}.

**Table 1:** Experimental Datasets with Different Charge State Assignment

Dataset	Charge state assignment	Number of peptides
C1	+1	212 (179/33)^a^
C2	+2	306 (267/39)
C3	+3	77 (66/11)

^a^In the bracket, the former is the number of peptides in the protein subset {P1}, and the latter is the peptides in the protein subset {P2}.

**Figure 1:**
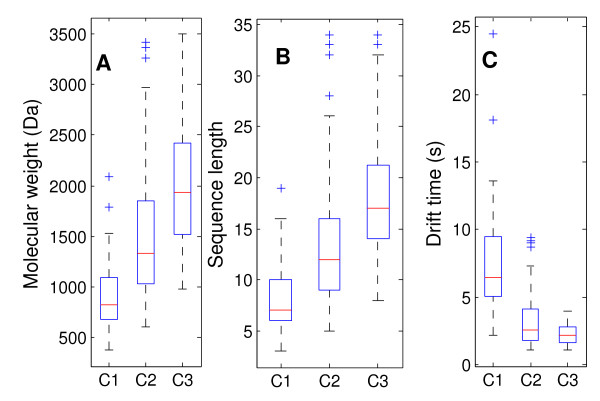
**Box plots of peptide molecular weight (A), sequence length (B) and drift time distribution (C) in the three datasets**. The central mark is the median, the edges of the box are the 25th and 75th percentiles, the whiskers extend to the most extreme data points that are not outliers, the cross points are outliers if they are larger than Q3+1.5*(Q3-Q1) or smaller than Q1-1.5*(Q3-Q1), where Q1 and Q3 are the 25th and 75th percentiles, respectively.

Figure 1 shows the distribution of peptide molecular weight, sequence length and drift time in each of the three datasets, i.e., C1, C2 and C3. The molecular weight distribution of peptides in each dataset has a relatively wide range from 374.22 Da to 3503.71 Da. The average molecular weight of the singly-charged peptides (dataset C1) is 900.14 Da, while the averaged molecular weight of the doubly-charged (dataset C2) and the triply-charged peptide ions (dataset C3) are 1470.39 Da and 2046.30 Da, respectively (Figure 1a). The number of amino acid residues in the 595 peptides ranges from 3 to 34. The average numbers of amino acid residues in these three datasets C1, C2 and C3 are 7.9, 13.2, and 18.3, respectively (Figure 1b). The distributions of peptide molecular weight and peptide sequence length in each dataset indicate that the large peptides, i.e., peptides with large molecular weight and long amino acid sequences, tend to have high charge states. The peptide ion drift time is also significantly related to the overall ion charge state. The mean value of peptide drift time for the singly-charged peptide ions is 7.48 s while the mean values of the doubly-charged and the triply-charged peptide ions are 3.07 s and 2.28 s, respectively (Figure 1c).

In this study, we developed our ANN regression models for the singly-, doubly-, and triply-charged peptide ions, respectively. During the ANN model construction, we employed a 10-fold cross-validation strategy [29]. For each of the three datasets, we first equally partitioned the entire dataset into 10 subsets in a random manner. Of the 10 subsets, a single subset was selected as validation data while the remaining 9 subsets were used to train the ANN model. The peptide drift times of the validation data were then predicted based on the trained ANN regression model. This process was repeated to ensure that every data subset was selected as the validation data for one time only. Therefore, 10 experiments were implemented for each charge specific dataset. By doing so, the drift time of each peptide ion in a dataset was predicted exactly once. The advantage of this cross-validation method is that all observations are used for both training and validation. This provides reliable learning of our model from the original data.

For a neural network model, the hidden layer configuration is very important because it introduces a nonlinear relationship into the network and provides the network with its ability to generalization. The model created in this paper is a back-propagation neural network model with a single hidden layer as increasing the number of hidden layers cannot improve the results [30]. However, choosing the number of nodes in the hidden layer is difficult because there are no acceptable theories to deal with this problem. It is generally accepted that selecting more nodes for the hidden layer will enable the model to "learn" more from the training data and have more power and flexibility. However, too many hidden nodes will increase the risk of over-fitting and an incapability of generalization [30]. Therefore, a balance between the learning ability and the generalization of the model must be investigated. Because of the complexity of the current problem and the number of nodes in the input and output layers, we first established a single-node hidden layer, and then the number of nodes in this hidden layer was increased iteratively by adding a single node in each iteration. We then chose an optimal number of the hidden layer nodes based on prediction results.

We employed the 10-fold cross-validation to find the optimal number of hidden layer nodes for our ANN model. The analysis was performed separately on each of the three charge-specific datasets, i.e., C1, C2 and C3. To a certain number of hidden layer nodes, we implemented the ANN regression model ten times. Table 2 shows the integrative performance of these repeated experiments under a prediction variation threshold of 15%. Here, prediction variation threshold was defined as the relative variation of the predicted peptide ion drift time from the experimentally observed drift time:(1)

**Table 2:** Prediction Accuracy as a Function of the Number of Hidden Layer Nodes under a Variation Threshold of 15% in Three Datasets

Hidden layer nodes	Prediction accuracy		
	
	C1	C2	C3
1	0.771 ± 0.053^a^	0.835 ± 0.059	0.712 ± 0.027
2	0.888 ± 0.023	0.936 ± 0.014	0.758 ± 0.028
3	0.944 ± 0.045	0.936 ± 0.009	0.742 ± 0.023
4	0.939 ± 0.023	0.929 ± 0.016	0.697 ± 0.020
5	0.922 ± 0.013	0.940 ± 0.004	0.742 ± 0.036

^a^The performance values consist of two measurements. One is the integrative prediction accuracy of five repeat experiments based on the variation threshold, and the other is the standard deviation of those accuracies.

where *η*_*pred *_is the prediction variation, *t*_*pred *_is the predicted peptide ion drift time and *t*_exp _is the experimentally observed peptide ion drift time.

Table 2 shows that the standard deviations of prediction accuracy of our ANN regression model range from 0.004 to 0.053 with an average value of 0.026. Such low values of the standard deviations in all of the repeated experiments, regardless of the dataset (C1, C2 and C3) and the number of hidden layer nodes, indicates the significant stability of our proposed ANN regression model. Table 2 also shows that the ANN regression model performs better when the hidden layer has multiple nodes. The prediction performance of the ANN model reaches a plateau and starts to fluctuate when the hidden layer contains more than 3 nodes. The best prediction accuracy for the singly-charged peptide ions (dataset C1) was obtained in three hidden nodes with a 0.944 accuracy value. The best performance of triply-charged peptide ions (dataset C3) was two hidden layer nodes with 0.758 accuracy value. The best performance of doubly-charged peptide ions (dataset C2) was five hidden layer nodes with 0.940 accuracy determination. However, such performance improvement from two nodes to five nodes is not significant, which is only about 0.004 accuracy improvement. It is well known that more hidden layer nodes may result in a higher computational cost and a higher probability of over-fitting [31]. Therefore, we chose a three-node hidden layer model for integrating the prediction performance in all of the three datasets.

**Figure 2:**
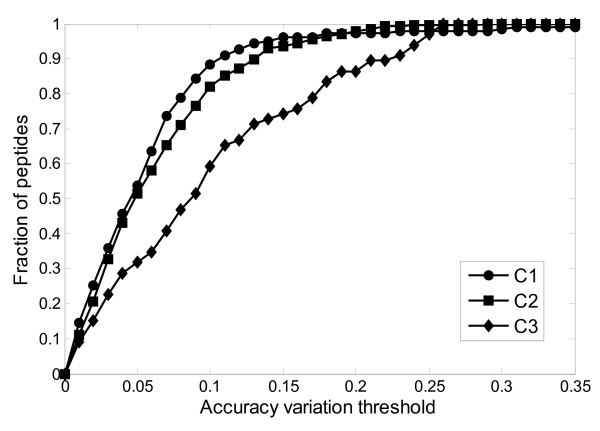
**The fraction of peptides vs. prediction accuracy variation threshold during the model construction process using the training dataset**. The diagram shows the number of peptides which can be predicted in different accuracy variation levels.

After determining the optimal three-node hidden layer, we used 10-fold cross validation to study the performance of our proposed ANN regression model on the training dataset. Figure 2 displays the relation between fractions of peptides with a correct prediction of drift time for the threshold of ANN prediction variation. Our proposed ANN regression model performs best on the singly-charged dataset. With 10% of prediction variation threshold, the ANN regression model correctly predicted the drift time of 88.3% of the singly-charged peptide ions. The fraction of correct prediction increases to 94.4% when the prediction variation threshold is set at 15%. The performance of the proposed ANN regression model on the doubly-charged peptide ions is close to its performance for the singly-charged peptide ions.

The performance of our model on the triply-charged peptide ions is relatively poor. With 15% prediction variation threshold, the ANN model can only correctly predict the drift time of 74% of the triply-charged peptide ions. The prediction accuracy increases to 95% if the prediction variation is set to 27%. We believe there are three reasons for such poor prediction accuracy of the triply-charged peptide ions. One is that the volume of the training dataset is not sufficient to obtain a reliable model. The training dataset contains only 66 triply-charged peptide ions, while there are 179 singly-charged peptide ions and 267 doubly-charged peptide ions. The other reason is that the four peptide features extracted from the peptide sequence do not include peptide conformation information. In general, the triply-charged peptides are large peptides. For example, the average peptide molecular weight of the triply-charged peptides in the training data is 2046.30 Da and the average peptide sequence length is 18.4 amino acid residues (Figure 1). Such large peptides usually form secondary structure, which will contribute to the peptide's cross-section and therefore, affect the peptide ion's drift time. Additionally, the larger coulomb force experienced by the triply-charged peptide ions may cause a larger range in overall cross-section differences; that is, many more species (notably shorter peptides) may adopt elongated conformations in order to minimize coulomb repulsion. This increased diversity in size distribution further compounds the problem of insufficient training dataset size. Unfortunately, a method of exactly predicting peptide ion conformation is not developed yet.

**Figure 3:**
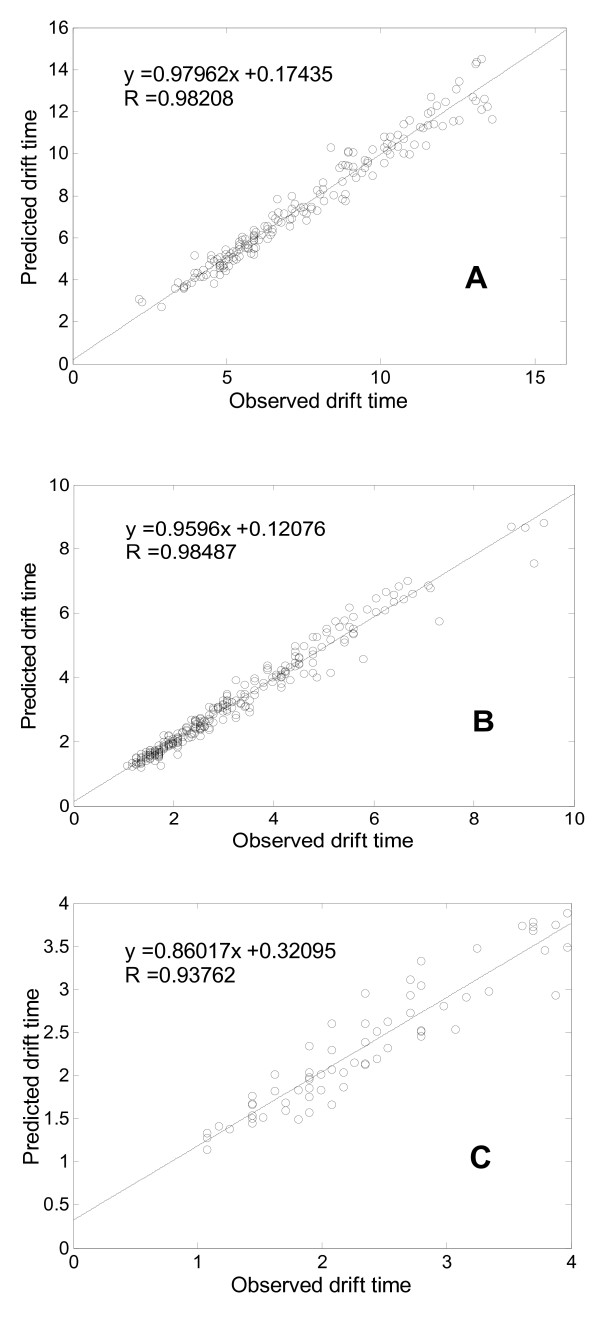
**Relationship between the observed and predicted drift times for the three peptide datasets**. Subfigures A, B and C are the regression results of our proposed ANN model for singly-, doubly-, and triply-charged peptide ions, respectively. The linear function in each subfigure is achieved by fitting the predicted results to observed drift times, and the line is the corresponding fitted curve. The correlation coefficients between observed and predicted peptide ion drift time are also shows in each subfigure.

Detailed regression results on the three training datasets can be found in Figure 3. The drift time distributions of peptide ions with different charge states show an evident variance. Most drift times of the singly-charged peptide ions range from 3 s to 14 s. The drift times of the doubly-charged peptide ions range from 1 s to 7 s, and the drift times of the triply-charged peptide ions range from 1 s to 4 s. The correlation coefficients between the predicted peptide ion drift time and the experimentally observed peptide ion drift time for the singly-, doubly-, and triply-charged peptide ions are 0.98, 0.98 and 0.94, respectively. Such high correlation signifies that our proposed ANN regression model can capture the general properties of peptide ion size for the prediction of drift time in IMMS measurements.

After constructing the ANN regression model, we used the testing dataset, i.e., peptides from the protein subset {P2}, to check its performance. Here the ANN regression model utilizes four nodes in the input layer, three nodes in the hidden layer and one node in the output layer. The testing dataset contains 82 peptides generated from the five randomly selected proteins {P2}. Overall there are 33 singly-charged peptide ions, 38 doubly-charged peptide ions and 11 triply-charged peptide ions. The prediction results of the ANN regression model on the testing dataset are listed in Table 3. With a 15% of prediction variation threshold, we achieved prediction accuracy of 0.909 for the singly-charge peptide ions, 0.872 for the doubly-charged and 0.727 for the triply-charged peptide ions. The mean difference between the experimental and the predicted values among the three charge states are 0.061, 0.008 and 0.128 ms, respectively. These prediction accuracies are very close to the performance of the ANN model obtained in the training dataset, which indicates our proposed model can be generalized well for the prediction of peptide ion drift time.

**Table 3:** Predicted Drift Times of Peptide ions in the Test Dataset Using an ANN Model with Three Hidden Layer Nodes

Peptide sequence			Mob_1+	Mob_1+ prediction	Mob_2+	Mob_2 + prediction	Mob_3+	Mob_3 + prediction
AQGHRPQDENPVVHFFK					4.24	4.44	2.26	2.17
FSWGAEGQKPGFGYGGR					3.97	3.96		
HRDTGILDSLGR					2.62	2.74	1.26	1.38
YLASASTMDHAR			11.4	11.83	2.8	2.57		
GLKGHDAQGTLSK					2.44	2.72		
TTHYGSLPQK			9.48	10.14	1.9	2.07		
FFGSDRGAPK					1.99	2.10		
DTGILDSLGR			8.75	9.02				
GHDAQGTLSK			8.48	8.69	1.71	1.86		
TPPPSQGK			5.51	6.69				
LGGRDSR					1.26	1.51		
FFGSDR			5.23	5.41				
HGFLPR			5.87	5.63	1.53	1.20		
SGSPMAR			4.69	5.51				
NIVTPR			5.42	5.36				
GLSLSR			4.78	4.74				
ASDYK			3.97	3.94				
NTDGSTDYGILQINSR					3.34	3.68		
WWCNDGRTPGSR					2.71	2.92		
FESNFNTQATNR					2.71	2.71		
GYSLGNWVCAAK			11.83	11.89	2.62	2.45		
GTDVQAWIR			8.12	8.96				
WWCNDGR			7.04	7.67	1.62	1.57		
HGLDNYR			6.5	7.01	1.53	1.49		
KVFGR			4.24	4.53	1.17	0.99		
TPGSR			3.16	3.76				
VFGR			3.43	3.39				
GCRL			3.34	3.44				
EPMIGVNQELAYFYPELFR					5.05	5.27		
FFVAPFPEVFGKEKVNELSK					4.87	5.17	2.08	2.64
HPIKHQGLPQEVLNENLLR					5.14	5.09	2.17	2.51
EDVPSERYLGYLEQLLR					4.33	4.43		
HQGLPQEVLNENLLR					3.7	3.67	1.81	1.90
FFVAPFPEVFGKEK					3.51	3.35		
FFVAPFPEVFGK			13.54	12.72				
HIQKEDVPSER							1.44	1.40
YLGYLEQLLR			12.55	11.27				
EKVNELSK								
TTMPLW			5.32	5.71				
TLTGKTITLEVEPSDTIENVK					5.42	5.24		
TLSDYNIQKESTLHLVLR					5.23	4.67	2.35	2.33
TITLEVEPSDTIENVKAK					4.61	4.29	2.17	2.20
TITLEVEPSDTIENVK			18.11	16.50	4.15	3.73		
IQDKEGIPPDQQR			13.45	13.04	2.44	3.02	1.26	1.54
LIFAGKQLEDGR			13.18	11.94	2.53	2.65	1.35	1.46
TLSDYNIQK			9.39	9.45	1.71	1.86		
ESTLHLVLR			9.3	9.21	1.99	1.91		
EGIPPDQQR			8.12	8.84	1.53	1.84		
MQIFVK			6.11	5.95	1.26	1.29		
QLEDGR			5.69	5.33	1.35	1.27		
LIFAGK			4.96	4.85	1.08	1.16		
TLTGK			3.16	3.62				
IQDK			3.02	3.11				
ADTIVAVELDTYPNTDIGDPSYPHIGIDIK							3.61	3.65
DQKDLILQGDATTGTDGNLELTR					6.23	5.75		
DLILQGDATTGTDGNLELTR					4.69	4.70		
VGLSASTGLYK					1.99	2.05		

The testing data were peptide digests of five randomly selected proteins. The tryptic digests of each protein were analyzed separately on IMMS. The drift time prediction performance for the peptide digest of each individual protein indicates the quality of prediction performance of our developed ANN regression model on different experiments operated under the same experimental conditions. The mean differences between the observed and the predicted drift time values of all tryptic peptide ions for the five proteins are 0.168, 0.206, -0.142, -0.039, and -0.091 ms, respectively. This small prediction difference among different protein digests, i.e., the different IMMS experiments, indicates our proposed prediction model is robust for the prediction of peptide ion drift time from repetitious experiments.

**Figure 4:**
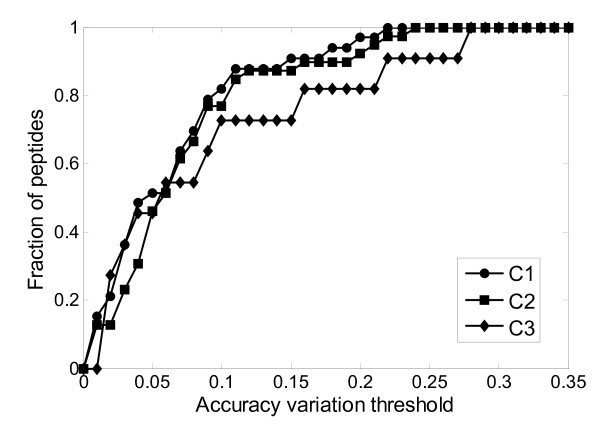
**The fraction of predicted peptides vs. prediction accuracy variation threshold on the testing data**.

Figure 4 shows the overall prediction performance of our proposed ANN regression model on the testing data. Our regression model has a similar performance for the drift time prediction of the singly- and the doubly-charged peptide ions. The prediction accuracy is poor for the triply-charged peptides. This is consistent with the results of our training dataset (Figure 2). In general, the prediction accuracy of our model on the testing dataset is slightly less than its prediction on the training dataset. For example, the prediction accuracy on the testing dataset is 0.909, 0.872 and 0.727 for C1, C2 and C3, respectively, while the prediction accuracy on the training dataset is 0.944, 0.936 and 0.742 using a prediction variation value that is less than 15%. This is understandable because the testing dataset did not contribute to the construction of the ANN regression model. On the other hand, the reduction of prediction accuracy is relatively small as also shown by the comparison of Figures 2 and 4, which demonstrates our proposed model has a reasonable degree of generalization performance.

It is well known that the prediction ability of an ANN depends on the model training. In the model training phase, there are two key elements. One is the quality of the original data; another is the architecture of model. These two facets are synergistically linked to each other. Because our original data is dependent on the original experimental environment, this model is applicable for the present experimental conditions. Under new conditions, our present model should be trained again and adjusted to the new states.

## Conclusions

In this study, an ANN regression model was developed to predict peptide ion drift time for IMMS measurements. To evaluate our proposed model, we tested its performance on a testing dataset which was not used during the model construction and training. The similar prediction accuracy between the training dataset and the testing dataset indicates the possibility of using the prediction results of the present model to assist protein identification efforts in proteomics studies. We achieved 94.4% prediction accuracy for +1 peptide ions and 93.6% for +2 ions. The relatively high level of performance indicated the capability of our proposed method. In addition, a simple net architecture consisting of four input nodes, three hidden nodes and one output node, makes our model more effective because the more simple net architecture is, the faster the ANN training and prediction will be. A relatively low prediction accuracy, 74.2%, for the +3 peptide ions suggests that spatial conformations of peptides with higher charge states presents an additional level of sample complexity that is not currently addressed in our current ANN model. Combining the conformation information, such as secondary structure formation, ion elongation, or interaction between peptide residues, into the present model will improve prediction capability and is the aim of future work. Furthermore, we plan to generalize the ANN model to predict the drift time of peptides with various post-translational modifications.

## Methods

### Samples preparation

20 proteins were purchased from Sigma Aldrich and used without further purification. A quantity (10 μg to 1 mg) of each protein was dissolved in 500 μL of denaturing solution (6 M urea in 200 mM ammonium bicarbonate, pH~8.0). To each protein sample, an aliquot of a stock solution of diothiothreitol (DTT, Sigma) was added such that the protein to DTT ratio (molar) was 1:40. The reduction reaction was allowed to proceed at 37°C for two hours. Next the samples were cooled on ice and an aliquot from a stock solution of iodoacetamide (IAM, Sigma) in 200 mM ammonium bicarbonate was added such that the protein to IAM ratio is 1:80. The reaction was allowed to proceed in darkness on ice for two hours. Then an aliquot of a cysteine stock solution in 200 mM ammonium bicarbonate is added to each sample such that the protein to cysteine ratio is 1:40. The quenching step was carried out at room temperature for 30 minutes. Next the sample solution was diluted with 200 mM ammonium bicarbonate buffer solution such that the final urea concentration was 2 M. Finally, an aliquot of a stock solution of trypsin (TPCK-treated, Sigma) in 200 mM ammonium bicarbonate was added to each sample such that trypsin was 2% of the protein content (by weight). The trypsin digestion was allowed to proceed for 24 hours at 37°C. Samples were desalted using solid phase extraction (Oasis HLB cartridges, Waters) and subsequently dried with a centrifugal concentrator (Labconco). Electrosprayed samples consisted of 1 × 10^-4 ^to 1 × 10^-2 ^mg of peptide digest dissolved in a water:acetonitrile:acetic acid (49:49:2) solution.

Peptide digest samples were analyzed by direct electrospray into the Synapt HDMS instrument (Waters). Each digest sample was infused through the electrospray needle at a flow rate of 5 μL·min^-1^. Dataset acquisition was carried out for a total of 3 minutes per sample. Peptide ions from each digest were separated in the T-wave instrument using a traveling wave height of 8.0 V and a speed of 300 m·s^-1^. The drift region of the Synapt was pressurized with 0.468 mBar of nitrogen gas. A total of 200 drift time bins were utilized in the work reported here. The duration of each bin corresponded to a single flight time distribution (0 to 2000 m/z for 250 μs) resulting in a drift time range of 50 ms. Flight time distributions were collected using the "V" reflectron mode of the Synapt instrument. Under these conditions, the typical resolving power and mass accuracy of the instrument were 10000 and 10 ppm, respectively.

Peptide ion assignments were obtained from a peptide mass fingerprint for each tryptic digest. The presence of a single component protein in each sample significantly increases the confidence of peptide ion assignments. The masses for dataset features were obtained based on m/z values and the isotopic spacing for each ion. These values were compared to theoretical peptide ions obtained from in-silico digests of proteins obtained from the Swiss-Prot protein database. Values of ± 0.02 Da were used as peptide ion assignment thresholds.

### Artificial neural networks

Many computational approaches have been proposed to analyze and process experimental data generated from MS for proteomics research [22,32]. Among these techniques, ANN-based methods are good choices for their capability of deriving useful information from complicated or imprecise data without the need of a detailed understanding of the underlying phenomena [23,24].

**Figure 5:**
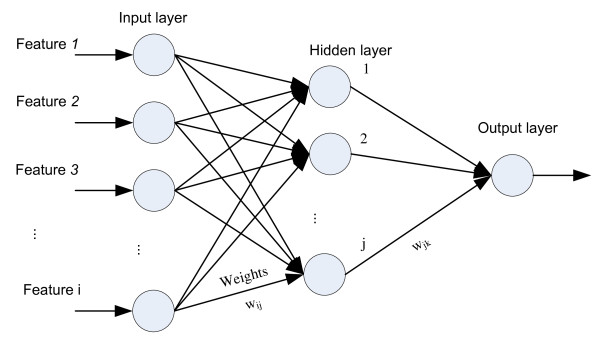
**A typical 3-layers neural network architecture**.

A typical architecture of an ANN contains an input layer, an output layer, and one or more hidden layers. In each layer, there are many nodes (nodes) which are connected with all or partial nodes in their previous and subsequent layer. In the ANN architecture, the nodes in the input layer receive the data and then the nodes in the hidden layer process and send them to the output layer. This process is conducted by weight which is the connection strength between two nodes. Each connection *j *→ *i *(from node *j *to node *i*) has a weight *w*_*ij *_that modulates the influence of node *j *on node *i *(Figure 5). The number of nodes in the input layer and the output layer are determined by the number of input and output parameters. The configuration of the hidden layer is usually constructed according to a given problem [33].

In this work, an ANN regression model based on a back-propagation algorithm was used to predict the drift times of peptides in IMMS. For each node in the hidden or output layer, its input value from the network was given by:(2)

where *w*_*ij *_is the weight from node *i*, *a*_*i *_is the activation of node *j*, and *θ *is the bias for node *i*. The activation of a node is determined by passing its net input through an activation function. Here, we adopted the logistic function:(3)

The goal of this network is to carry out a desired input-output mapping. The learning process of an ANN is to adjust the weights of the connections between nodes of different layer to an optimum set of values for this mapping. After training processes were finished, the ANN can be applied to the prediction task.

### Peptide representation

To generate a predictor that can infer peptide ion drift time in IMMS using an ANN regression method, each peptide ion must be represented as an n-dimensional vector in a given feature space. Three features were extracted from the peptide sequence: the molecular weight, the sequence length and the sequence index. The molecular weight feature is the sum of the molecular weight of each amino acid residue. The sequence length feature denotes the number of residues contained within the peptide. However, the molecular weight and the sequence length can not describe the amino acid sequence-order information of a peptide. The sequence index therefore was designed to reflect the influence of the order of the amino acids in the peptide sequence, which is similar to our previous work [23]. In order to distinguish peptides more effectively, we extended the previous sequence index into a two-dimensional form:(4)

where *m*_*i *_is the ionization constant (pKa) value of the *i*-th amino acid residue in the peptide, and *N *is the sequence length of the peptide. The pKa value of each amino acid residue was derived in the same way as our former work [23,34].

Therefore, each peptide can be represented by the three features using a four-dimensional vector as following:(5)

where *p *denotes the target peptide, *mw *molecular weight, *sl *sequence length, and *si*_1_, *si*_2 _denote the sequence index.

### Feature normalization

Based on the peptide features we used here, each peptide has been represented by a four-dimensional feature vector consisting of 1-D molecular weight, 1-D sequence length and 2-D sequence index. However, the features are derived from different facets of peptide sequence and have different units, which will bring an imbalanced expression level among peptide features. This results in a variation in contribution of each peptide feature to the predictor performance. Therefore, the feature values must be normalized to equally reflect (as much as possible) the influence of each feature.

For a given peptide dataset *P *= (*p*_1_, *p*_2_,..., *p*_*N*_), it can be represented by a 4 × *N *matrix:(6)

Every feature value of each category, also each row in the above formula, was normalized using the formula:(7)

where *f *is the raw value of feature *mw*, *sl*, *si*_1 _and *si*_2_, *f*_*normalized *_denotes the normalized value of this feature, *f*_min _and *f*_max _are the minimum and maximum values of the corresponding feature category. After normalization, all values of each feature always fall within a fixed interval [-1, 1].

### Model construction and training

The ANN regression model used in this study consisted of three layers (i.e., the input layer, the hidden layer and the output layer). Each peptide was encoded as a four-dimensional vector using the three features mentioned above. The input layer of our ANN model consisted of four nodes; the output layer only had one node. Additionally, the hidden layer configuration for the model was determined empirically by the number of nodes in the input and the output layers. We tested the number of hidden nodes from 1 to 5, and chose an optimized value by which our model achieved the best performance.

## Authors' contributions

BW and XZ conceived of the study; SV, MP, and SR participated in the experiment design; BW, SV and XZ carried it out and drafted the manuscript. All authors read and approved the final manuscript.
